# Uterine Leiomyoma with Massive Lymphoid Infiltrate in a Patient with History of Assisted In-Vitro Fertilization

**DOI:** 10.5146/tjpath.2019.01476

**Published:** 2020-09-15

**Authors:** Monia Di Prete, Matteo Collamarini, Claudio Collamarini, Francesco Sesti, Alessandro Mauriello, Giampiero Palmieri

**Affiliations:** Department of Anatomic Pathology,University of Rome Tor Vergata, Rome, Italy; Department of Gynaecology and Obstetrics, University of Rome Tor Vergata, Rome, Italy

**Keywords:** Leiomyoma, In-vitro fertilization, Uterine, Lymphoma

## Abstract

Uterine leiomyomas are the most common benign tumors of the gynecological tract. Massive lymphocytic infiltration has been reported rarely in uterine leiomyomas and it has been described as a pathogenetic correlation with gonadotropin-releasing hormone agonists. Uterine leiomyomas with massive lymphoid infiltration have to be differentiated from non-Hodgkin lymphomas. We report a case of a woman without a history of gonadotropin-releasing hormone agonist treatment, who presented with a uterine leiomyoma that increased in size after the procedure of assisted in-vitro fertilization, and associated with massive nodular lymphoid infiltrate simulating, morphologically, a non-Hodgkin lymphoma. Uterine leiomyoma with massive lymphocytic infiltration is a very rare entity, probably of reactive significance, which has to be differentiated from diseases that need a systemic therapeutic approach.

## INTRODUCTION

Uterine leiomyomas are the most common benign tumors of the gynecological tract and occur in at least 20% of women in their reproductive age (1). Lymphoid infiltration involving the uterus is generally confined to the cervix (2) but massive lymphocytic infiltration has also rarely been reported in uterine leiomyomas (3–10). A pathogenetic correlation between lymphoid infiltration and treatment with gonadotropin-releasing hormone (GnRH) agonists has been described in literature, even if the mechanism is still unclear (6,7). GnRH agonists have been used in the management of uterine leiomyomas, as a neoadjuvant therapy before surgical excision, since the 1980s (11). The treatment produces size reduction, which results in an easier and more conservative surgical approach (myomectomy instead of hysterectomy, or vaginal rather than abdominal hysterectomy). The underlying mechanism is based on decreasing the production of luteinizing and follicle-stimulating hormones from the pituitary gland, which induces a subsequent hypoestrogenic status. Uterine leiomyomas are estrogen-dependent tumors and the decreased estrogenic stimulation leads to a reduction in size (12). Uterine leiomyomas with massive lymphoid infiltration have to be differentiated from non-Hodgkin lymphomas (NHL). We report a case of a thirty-seven-year-old woman, who had undergone assisted *in-vitro* fertilization, without using GnRH agonists, and who presented a uterine leiomyoma with massive nodular lymphoid infiltrate simulating NHL.

## CASE REPORT

**Figure 1:**
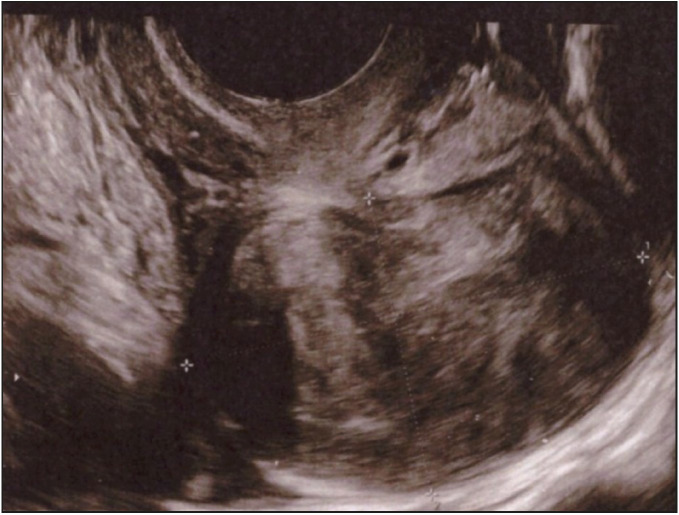
Transvaginal ultrasound shows a well-defined, solid subserosal mass, with variable echogenicity, that causes acoustic shadowing, suggestive for leiomyoma.

**Figure 2:**
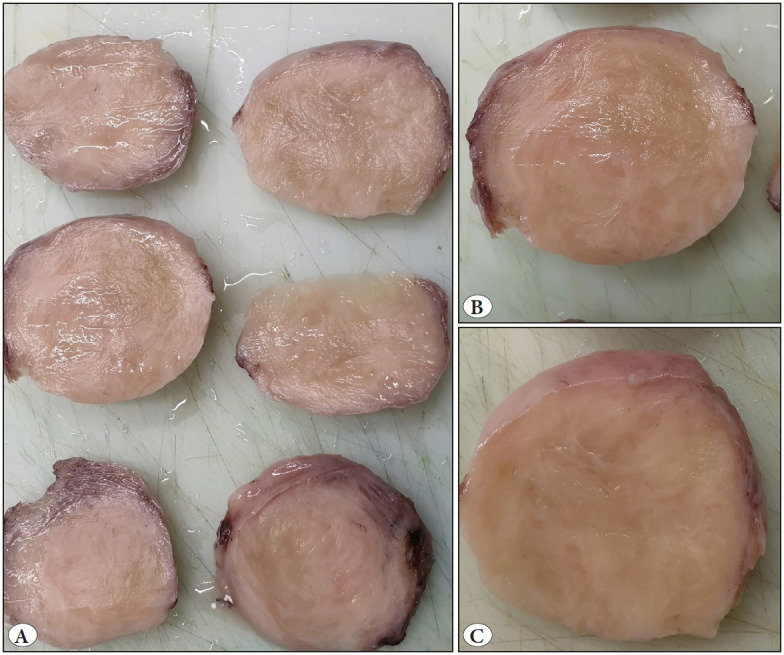
**A)** Whitish ovoidal formation of firm consistency and smooth surface. **B-C)** The cut surface reveals typical interlacing bundles, with no areas of necrosis, hemorrhage or softening.

**Figure 3:**
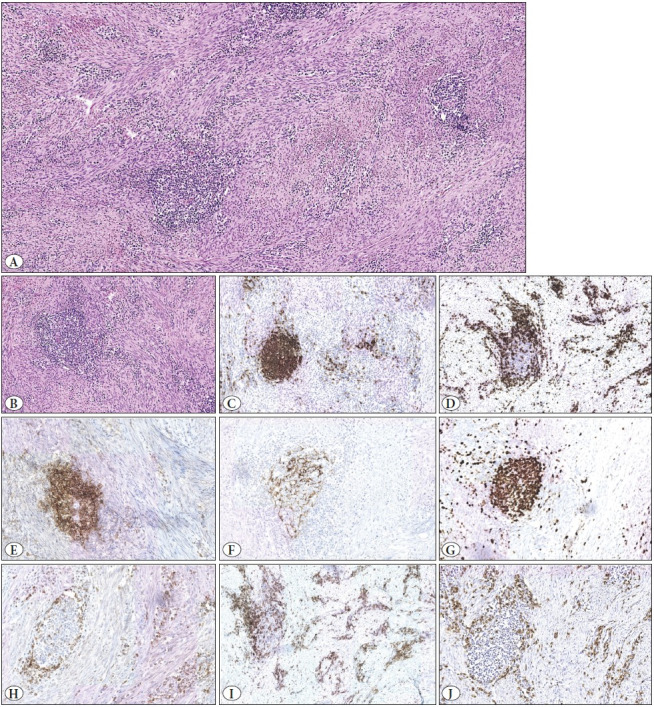
**A)** Microscopic examination shows bland smooth-muscle spindle cells, arranged in fascicles, associated with a dense lymphoid infiltration of small and mature lymphocytes, organized in nodules alternated with diffuse areas (H&E; 100). **B-J)** The immunohistochemical study of a lymphoid follicle. **C)** CD20. **D)** CD3. **E)** CD10. **F)** CD21. **G)** Ki-67. **H)** bcl-2. **I)** CD4. **J)** CD8. (H&E; x100) (IHC; x100).

A 37-year-old Caucasian woman was referred to our Gynecological Department for a slowly-growing leiomyoma of the posterior wall of the uterus. She had no recent history of uterine bleeding, abdominal pain or drug assumption. A transvaginal ultrasound scan showed no evidence of endometrial hyperplasia. Pap smear was negative for malignancy and laboratory tests showed no significant alterations. The patient had undergone two cycles of assisted *in-vitro* fertilization, by intra-cytoplasmic sperm injection (ICSI), three years ago, when a subserosal fibroid 1 cm in diameter was first described. During the fertilization procedure, she took Cetrotide, a GnRH antagonist. She had one pregnancy, with subsequent natural eutopic delivery. The myoma grew in size rapidly after the fertilization procedure and pregnancy was checked periodically (Figure 1) until it was decided to perform a myomectomy. The postoperative course was uneventful. Grossly, the specimen consisted of a whitish ovoidal formation, measuring 6 cm in greatest dimension, of firm consistency and smooth surface. The cut surface showed a typical aspect of interlacing bundles, with no areas of necrosis, hemorrhage or softening (Figure 2). Microscopic examination revealed a well-circumscribed leiomyoma composed of bland smooth-muscle spindle cells arranged in fascicles, associated with a dense lymphoid infiltration of small and mature lymphocytes organized in nodules (Figure 3). These areas alternated with others where the infiltrate was diffuse but less dense. A few plasma cells were identified . Occasional larger lymphocytes were present. Neither tangible-body macrophages nor neutrophils were detected. Vascularity was not prominent with thin-walled blood vessels lined by flattened endothelium. Although the density of the lymphoid infiltrate was sometimes greater around the blood vessels, pathogens, fibrin deposits or thrombi were absent in the tumor vessels. The massive lymphoid infiltration imposed the differential diagnosis with NHL. The nodular infiltrate consisted of lymphoid follicles with germinal center consisting of CD20 + (with a few small T lymphocytes CD3 + interspersed), bcl-2 -, CD10 +, bcl-6 + associated with a meshwork of follicular dendritic cells CD21 +. The Ki67 proliferation index was high in reactive germinal centers. Small lymphocytes with diffuse growth pattern were predominately CD3 + and CD4 +, with a CD4 + : CD8 + ratio of 3 : 1. Immunohistochemical findings are shown in Figure 3. Only occasional scattered lymphocytes were positive for granzyme B. Moreover, the immunohistochemical staining for ALK-1 was negative, excluding an inflammatory myofibroblastic tumor. This finding, associated with the polymorphic composition of the infiltrate, led to the conclusion of a leiomyoma with massive lymphoid infiltration.

## DISCUSSION

Uterine leiomyoma with lymphoid infiltration is a very rare condition. It is characterized by a small lymphocyte infiltrate that can be focal to diffuse, moderate to brisk in density, and associated with a few plasma cells, rare eosinophils and occasional lymphoblasts (3,4). Germinal centers can be present but generally are not prominent. The infiltrate is generally composed predominately by small and mature CD8 + T-lymphocytes. The underlying causes are not yet identified. The association with previous treatment with GnRH agonists has been reported in the literature (6,7). It was suggested that the pathogenic mechanism derives from the immunological response elicited against cell surface antigens altered by the therapy (13). In our case, the patient underwent an ICSI procedure, which took place three years before the myomectomy. Fibroids’ dramatic enlargement can be related to ICSI and pregnancy, as reported in the literature (14). Moreover, our patient took Cetrotide, a GnRH antagonist, which was not previously associated with uterine leiomyoma lymphoid infiltration, during the procedure. GnRH antagonists, as the agonists, are routinely used as a neoadjuvant treatment before surgical removal of fibroids. These considerations lead to the hypothesis that the lymphoid infiltrate, detected in our case, may be connected with the therapy administered to our patient (13). The lymphocytic infiltration, composed predominantly of small and mature lymphocytes and organized in lymphoid follicles with a germinal center, is the peculiar feature of our case. Massive lymphoid infiltration in uterine leiomyomas has also been reported in association with intrauterine contraceptive devices or autoimmune diseases. The former may cause episodes of acute inflammation within the myoma (3). The latter may elicit a direct cytotoxic effect (15). A recent study on Hodgkin’s disease and anaplastic large cell lymphoma reported that tumor cells may aberrantly express TNF-*α* or thymus- and activation-regulated chemokines (TARC), which might attract a specific lymphocytic subset and CD4 + T-cell with a Th2 phenotype in particular (16). Unfortunately, we could not perform immunohistochemical staining for TNF-*α* and TARC. Malignant tumors can be infiltrated by T-lymphocytes (TILs: tumor-infiltrating lymphocytes) due to a specific host response to the tumor itself (17). However, to our knowledge, TILs are described only in association with malignant tumors. The differential diagnosis of uterine leiomyoma with lymphoid infiltration includes uterine malignant lymphomas and sarcomas, such as an inflammatory myofibroblastic tumor, as well as reactive conditions. Uterine leiomyoma with massive lymphoid infiltration cannot be distinguished from NHL by clinical features; the latter may not have a systemic presentation and can be confined to the uterus, even involving preexistent fibroids (3,18). The gross appearance of leiomyomas with and without lymphoid infiltration is similar, while malignant lymphoma is softer and fleshy. On histologic examination, the uterus, including the cervix, is generally involved by diffuse large B-cell lymphoma (18). In our case, the lymphoid infiltrate was composed by small lymphocytes arranged in follicles, with a germinal center, that was focally prominent, enforcing the differential diagnosis with follicular lymphoma. This type of malignant lymphoma is extremely rare in the uterus, and the cellular composition (presence of plasma cells) associated with the irregularity in size and shape of the lymphoid follicles, as evidenced by immunohistochemistry, led to the diagnosis of a leiomyoma with lymphoid infiltration. It was not possible to evaluate the surrounding tissues, but the fibroid was well-circumscribed and the lymphoid infiltration appeared to be confined to the lesion, while malignant lymphoma generally spreads to the adjacent myometrium. Another differential diagnosis that has to be considered is inflammatory myofibroblastic tumor, which is generally positive for ALK-1 (19). In our case, the immunostaining was negative and the background cells did not express a myofibroblastic immunophenotype.

In conclusion, uterine leiomyoma with massive lymphocytic infiltration is a very rare entity, probably of reactive significance, and has distinct morphological features, which allow the differential diagnosis from diseases that need a systemic therapeutic approach such as NHL.

## Conflict of Interest

The authors declare no conflict of interest nor funding sources.
